# Ballet foot in a boy

**DOI:** 10.1111/1756-185X.14106

**Published:** 2021-04-07

**Authors:** Teresa Giani, Maurizio Gattinara, Rolando Cimaz

**Affiliations:** ^1^ Department of Medical Biotechnology University of Siena Siena Italy; ^2^ ASST G.Pini‐CTO Milano Italy; ^3^ Department of Clinical Sciences and Community Health University of Milano Milano Italy

**Keywords:** clinical aspects, rehabilitation

A previously healthy 10‐year‐old boy presented with a 2‐month history of pain and marked non‐reducible plantar flexion of the right foot. Symptoms occurred after a mild injury to his right foot and progressively had increased during the last month, persisting all day long, and worsening with movements, limiting daily activities including school attendance. Prolonged bed rest, and treatment with different types of non‐steroidal anti‐inflammatory drugs was ineffective. A similar, but milder and transient episode had occurred a year earlier. His family history was unremarkable.

On physical examination the patient presented in good general health. His right foot was in a fixed posture, with non‐reducible plantar flexion of ankle and toes (Figure 1A). Mild swelling was palpable on the dorsal area of the foot with preserved skin color. His ankle joint mobility was completely restricted, and the boy was unable to bear weight on the affected side. Extreme sensitivity to light touch was appreciable, and pain assessment by visual analog scale was referred by the child to be 8 out of 10.

- What is the possible diagnosis?
- Which test can confirm the diagnosis?
- What is the first treatment step?

**FIGURE 1:**
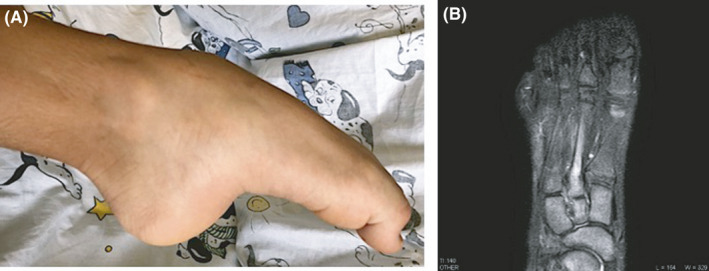
(A) Clinical aspect of right foot, which is kept in forced full plantar flexion. Ankle, midfoot, and toes are contracted, and the flexion is non‐reducible for marked opposition by the child. Under general anesthesia the contracture could partially be reversed, but returned to original position upon awakening. (B) Magnetic resonance imaging of the foot showing bone marrow edema of second metatarsal bone

**Answers on page 2.**

## 1 “BALLET FOOT” IN A BOY: PART 2

### 1.1 What is the possible diagnosis?

- Complex regional pain syndrome (CRPS) type I triggered by a minor, accidental injury.

### 1.2 Which test can confirm the diagnosis?

- CRPS is a clinical diagnosis, there is no specific single test to confirm it. In this boy, as expected, routine laboratory exams resulted within normal range.

Classic radiography may support the diagnosis with suggestive but non‐specific features such as soft tissue swelling, periarticular, and patchy osteopenia; magnetic resonance may add some information as bone marrow edema, muscle atrophy, joint effusion and synovial hypertrophy may be present. In this patient axial short tau inversion recovery magnetic resonance imaging of the right foot showed a bone marrow edema at the right second metatarsal (Figure 1B).

### 1.3 What is the first treatment step?

- Treatment should provide an early multidisciplinary management, initially based on physical/occupational, and psychological therapy and on educational strategies.

In our case multiple cycles of physical therapy, massage, transcutaneous electrical nerve stimulation and mobilization, as well as different pharmacological treatments including gabapentin, amitriptyline, and pamidronate were unsuccessfully tried. Due to the persistence of the pain and the impossibility to return to routine daily activities, we treated him with parenteral ketamine; however, after 5 infusions (1 mg/kg) only a partial benefit was obtained. Only after 3 days of continuous sciatic nerve block, an initial, clear improvement was achieved. Immediately a tight, and well‐coordinated multidisciplinary meeting program involving neuropsychiatrist, physical therapist, pediatric rheumatologist, and anesthesiologist was organized. The boy gradually returned to school over a 2‐month period.

**Key learning points**

- CRPS is part of the larger pediatric chapter defined as amplified musculoskeletal pain syndromes.
- CRPS type I follows days to a month after trauma (often of mild entity), in the absence of nerve injury.
- Distal extremities are the typically involved sites.
- Diagnosis is one of exclusion, it is mostly based on clinical signs such as disproportionate pain intensity and duration, allodynia, autonomic disturbances, and severe disability.^1^
- Magnetic resonance may show skin thickness, subcutaneous edema, muscle atrophy, bone marrow edema and, in chronic cases, bone mineral loss.
- Treatment requires a multidisciplinary, and patient‐centered approach.^2^, ^3^
- Second line treatments such as bisphosphonates, opioids, botulinum toxin injections, ketamine, or sympathetic nerve block should be considered in severe, unresponsive cases.^4^, ^5^
